# The Effect of Oxycodone on Post-operative Pain and Inflammatory Cytokine Release in Elderly Patients Undergoing Laparoscopic Gastrectomy

**DOI:** 10.3389/fmed.2021.700025

**Published:** 2021-09-01

**Authors:** Wei-long Lao, Qi-liang Song, Zong-ming Jiang, Wen-di Chen, Xian-he Zheng, Zhong-hua Chen

**Affiliations:** ^1^Shaoxing University School of Medicine, Shaoxing, China; ^2^Department of Anesthesia, Shaoxing People's Hospital, Shaoxing, China; ^3^Department of Anesthesia, The First Affiliated Hospital of Shaoxing University, Shaoxing, China

**Keywords:** oxycodone, post-operative analgesia, inflammatory cytokine, laparoscope, gastric cancer

## Abstract

**Background:** To evaluate the effect of oxycodone on post-operative pain and inflammation in elderly patients undergoing laparoscopic gastrectomy.

**Methods:** Sixty patients who were of both sexes, American Society of Anesthesiologists Physical Status (ASA-PS) Class I or II, over 65 years of age and undergoing an elective laparoscopic radical gastrectomy were randomly divided into two groups: an oxycodone group (Group O) including 20 males and 10 females and a sufentanil group (Group S) including 21 males and 9 females. The post-operative analgesia regimen was as follows: 40 mg of parecoxib sodium and 0.1 mg/kg of oxycodone was intravenously injected into Group O before the abdomen closure, while 40 mg of parecoxib sodium and 0.1 μg/kg of sufentanil was injected intravenously into Group S. Both groups were infiltrated with 20 ml of 1% ropivacaine at the end of the operation. The level of serum IL-6 and IL-10 were assayed immediately at the following timepoints: at the conclusion of surgery (T1), 1 h (T2), 6 h (T3), and 24 h (T4) after the completion of the surgery. The numerical rating scale (NRS), the Ramsay sedation score, analgesic-related adverse events, post-operative pulmonary inflammation events and the post-operative stay were recorded.

**Results:** Compared with Group S, the serum IL-6 concentrations of Group O decreased at T_3_ and T_4_, while the serum IL-10 concentrations increased (*P* < 0.05). In Group O, the serum IL-6 concentrations at T_3_ and T_4_ were lower than those at T_1_ (*P* < 0.05). The incidence of post-operative nausea and vomiting (PONV) and pulmonary inflammation in Group O was lower than that in Group S (*P* < 0.05). At each time point, the NRS of visceral pain in Group O was lower than that in Group S. At 6 and 24 h after extubation, the NRS of incision pain in Group O was lower than that in Group S (*P* < 0.05).

**Conclusion:** Oxycodone can regulate the level of inflammatory cytokines and reduce post-operative inflammatory response.

## Background

Gastric cancer is still the fourth most common cancer in the world, and its mortality rate is the second highest cancer mortality rate (1). So far, many studies have proven the safety of laparoscopic surgery for gastrointestinal diseases. One example is Zheng Lijun et al.'s retrospective comparative study of laparoscopic and open distal gastrectomy in the treatment of elderly gastric cancer. Their study, using relevant data from a number of years, shows that a laparoscopic radical gastrectomy is effective and safe for treating gastric cancer and may be superior to the traditional open gastrectomy with respect to some of its surgical effects (2).

Because of the particular physical condition of elderly patients, it is extremely important to attention post-operative adverse reaction. Post-operative pain is a common adverse reaction of elderly patients after a laparoscopic radical gastrectomy for gastric cancer. Poor analgesia will cause a severe stress reaction and adversely affect post-operative recovery. According to previous studies, these adverse effects mainly include decreased vital capacity and alveolar ventilation, pneumonia, tachycardia, hypertension, myocardial infarction and myocardial ischemia (3–5), and noxious stimulation during the operation will increase the release of pro-inflammatory factors and reduce the release of anti-inflammatory factors (6). Serum IL-6 is a cytokine with inflammation-mediated activity, which reflects the degree of tissue injury and post-operative stress (7) while serum IL-10 is a potent immunosuppressive cytokine, which can inhibit proinflammatory cytokines (8), resulting in systemic inflammation and affecting post-operative recovery. Therefore, for the elderly patients, the selection of appropriate analgesic is very important. Opioids are the first choice for post-operative analgesia (9). Nausea and vomiting is a common side effect of opioids, and the μ receptor is the main receptor that causes nausea and vomiting. Sufentanil, an opioid analgesic, is often used as a pure μ receptor agonist, and we often increase its dose to avoid the occurrence of analgesia deficiency. However, it also increases the incidence of post-operative nausea and vomiting (10). Oxycodone is a semi-synthetic opioid analgesic, which can effectively relieve visceral pain by stimulating μ and κ receptors, especially κ receptors, and it has fewer adverse reactions than other opioid drugs (11, 12). Studies have shown that both μ and κ receptors exist in the gastrointestinal tract, and their functions include controlling visceral pain (13). In view of the κ receptor agonist effect of oxycodone, its analgesic effect on visceral pain is better than that of the μ receptor agonist alone (12, 14).

Although recent clinical research into oxycodone has involved analgesia in many fields, there is no research into the analgesic and anti-inflammatory effects of oxycodone in elderly patients after a radical gastrectomy for gastric cancer. This study aims to evaluate the effect of oxycodone hydrochloride on post-operative pain and inflammation in elderly patients undergoing a laparoscopic radical gastrectomy for gastric cancer, so as to provide a reference for clinical research.

## Methods

### Subjects

**Figure 1:**
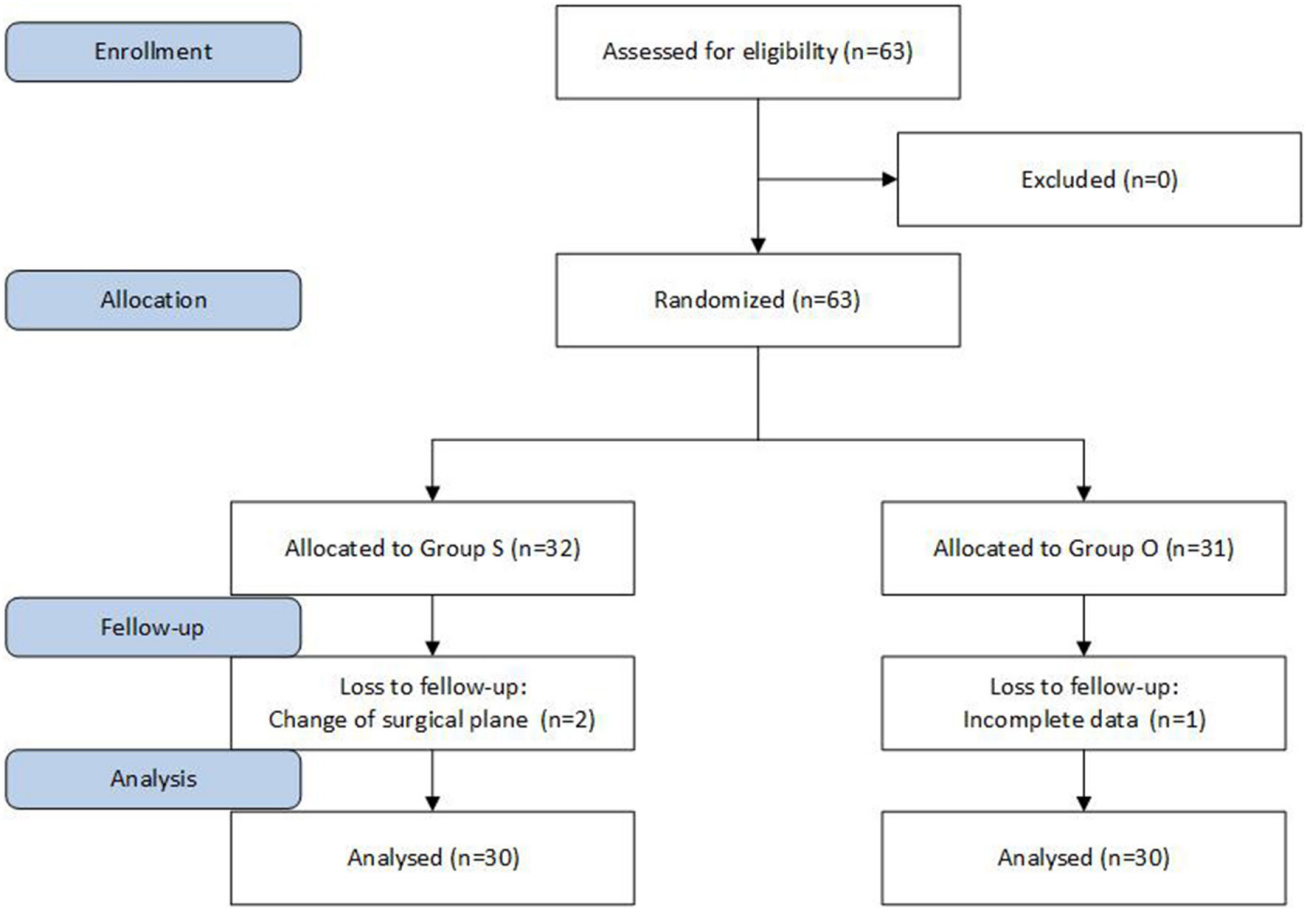
Summary of protocol.

This study was approved by the Ethics Committee of our hospital, and the informed consent of patients and their families was obtained. The subjects had undergone a laparoscopic radical resection for gastric cancer; there were 60 cases, aged 65 or above, ASA-PS I or II, male and female. The patients had no neurological or psychiatric problems, no long-term use of sedatives or antidepressants, and no history of alcohol abuse or drug dependence. Patients with pre-operative liver and kidney abnormalities and chronic pain, neurological or psychiatric disorders, long-term use of sedatives and painkillers and long-term use of antipsychotic medications were excluded. The patients were divided into an oxycodone group (Group O) and a sufentanil group (Group S) according to the random number table method, with 30 patients in each group (Figure 1).

### Protocol

There was pre-operative routine abstinence from drinking and fasting and no pre-operative medication. After the patient was admitted to the operating room, routine ECG monitoring was performed to establish the peripheral venous access of the upper limb, and 500 ml of Ringer's lactate solution was given (20–25 min). A left radial artery puncture and catheterization were performed under local anesthesia to monitor the invasive blood pressure, and a BIS monitor was connected to monitor the anesthesia depth. Anesthesia was induced with an intravenous injection of midazolam 0.02 mg/kg, etomidate 0.2–0.3 mg/kg, sufentanil 0.6 μg/kg, and cis atracurium 0.3 mg/kg. After endotracheal intubation, mechanical ventilation was performed. The tidal volume was 8:10 ml/kg, the respiratory frequency was 12:14 bpm, the inspiratory/expiratory ratio was 1:2, the end-expiratory carbon dioxide pressure was 35–40 mmHg, the oxygen flow was 2 L/min, and the BIS was 45–55. Anesthesia was maintained with an intravenous constant speed pump infusion of remifentanil 0.1–0.3 μg·kg^−1^·min^−1^, a target-controlled infusion of propofol (serum target concentration 2.5–3.0 μg/ml) and the intermittent addition of cis atracurium to maintain muscle relaxation. Post-operative analgesic methods: Once the abdomen was closed, group O was given 40 mg parecoxib sodium and 0.1 mg/kg oxycodone. Group S was administered 40 mg parecoxib sodium and 0.1 g/kg sufentanil. Both groups received 1% ropivacaine 20 ml incision infiltration at the end of the operation.

Venous blood samples were collected immediately before the operation (T1) and 1 h (T2), 6 h (T3), and 24 h (T4) after the operation. Serum IL-6 and IL-10 concentrations were determined by an ELISA blood test. The digital pain score (NRS) (0 for no pain, 10 for the most pain, 1–3 for mild pain, 4–6 for moderate pain, and 7–10 for severe pain) was recorded at 1, 5, 10, and 30 min and 1, 6, 24, and 48 h after extubation. Analgesic drugs (Sufentanil, parecoxib sodium, etc.) were administered to all patients with an NRS >4. The Ramsay score was recorded at 1, 5, 10, 30, and 60 min after extubation. The time of resuscitation (from the end of the operation to return to the ward), the incidence of analgesia-related adverse events (nausea, emesis, respiratory depression, restlessness during the waking period), post-operative pulmonary inflammation and post-operative hospital stay were all recorded.

### Statistical Analysis

SPSS 22.0 statistical software was used for analysis. Measurement data of normal distribution were expressed as mean ± standard deviation ($\bar{\text{x}}$ ± s), a group *T*-test was used for inter-group comparison, and repeated measurement analysis of variance was used for intra-group comparison. Count data were compared using the chi-square test or Fisher exact probability calculations. *P* < 0.05 was considered statistically significant.

## Results

### General Conditions

A total of 60 patients were included in this study, randomly divided into two groups: the group O including 20 males and 10 females and the group S including 21 males and 9 females. There was no statistically significant difference in general conditions between the two groups (*P* > 0.05), as shown in Table 1.

**Table 1:** Comparison of general conditions between the two groups.

**Group**	**Group O (*n* = 30)**	**Group S (*n* = 30)**	***P-*value**
Gender (male/female)	20/10	21/9	0.781
Age (years old)	71.8 ± 9.6	72.9 ± 9.2	0.652
Height (cm)	164.2 ± 7.0	163.8 ± 8.0	0.810
Weight (kg)	59.2 ± 9.4	58.6 ± 10.2	0.840
Surgical type for gastric cancer			0.301
Total gastrectomy	4	4	
Distal gastrectomy	6	8	
Curative gastrectomy	15	12	
Remnant gastrectomy	5	6	

### IL-6 and IL-10 Concentrations at Each Period

There was no significant difference in serum IL-6 and IL-10 concentrations at T1 and T2 between the two groups (*P* > 0.05). Compared with Group S, serum IL-6 concentration decreases and IL-10 concentration increases at T3 and T4 in Group O. In group O, the serum IL-6 concentration at T3 and T4 was lower than it was at T1, with a statistically significant difference (*P* < 0.05), as shown in Table 2.

**Table 2:** Comparison of serum IL-6 and IL-10 concentrations between the two groups.

**Indicator**	**IL-6 (pg/ml)**			**IL-10 (pg/ml)**		
**Group**	**Group O (*n* = 30)**	**Group S (*n* = 30)**	***P-*value**	**Group O (*n* = 30)**	**Group S (*n* = 30)**	***P-*value**
T_1_	158.8 ± 26.7	155.4 ± 27.0	0.628	14.6 ± 3.8	14.2 ± 3.3	0.676
T_2_	145.5 ± 27.0	158.3 ± 24.1	0.058	16.0 ± 4.0	14.5 ± 3.3	0.120
T_3_	142.8 ± 26.7^a^^b^	162.6 ± 21.7	0.003	15.7 ± 3.7^b^	13.2 ± 3.4	0.010
T_4_	141.9 ± 24.8^a^^b^	159.3 ± 26.3	0.011	15.3 ± 4.0^b^	13.1 ± 3.7	0.028

*Compared with T1*,

a *P < 0.05; Compared with Group S*,

b *P < 0.05*.

### Post-operative Situation

There were no statistically significant differences between the two groups in post-operative respiratory depression, incidence of agitation in the wake period, resuscitation time and post-operative hospital stay (*P* > 0.05). The incidence of post-operative nausea, vomiting and pulmonary inflammation in group O was lower than that in group S, with statistically significant differences (*P* < 0.05), as shown in Table 3.

**Table 3:** Comparison of adverse events related to analgesia, pulmonary inflammation and post-operative hospital stay between the two groups [cases (%)].

**Group**	**Group O**	**Group S**	***P-*value**
Nausea,%	3 (10.0)^a^	11 (36.7)	0.015
Emesis (%)	1 (3.3)^a^	7 (20.0)	0.026
Respiratory depression	0 (0)	0 (0)	
Restlessness during the waking period	1 (3.3)	3 (10.0)	0.306
Awakening time	68.3 ± 10.5	64.5 ± 10.4	0.164
Occurrence of pulmonary inflammation	5 (16.7)^a^	12 (40.0)	0.042
Post-operative hospital stay	15.1 ± 6.9	15.0 ± 4.2	0.912

*Compared with Group S*,

a *P < 0.05*.

### Scores

At each time point, the NRS score in group O was lower than that in group S. At 6 and 24 h after surgery, the NRS score of incision pain in group O was lower than that in group S, with a statistically significant difference (*P* < 0.05), as shown in Table 4. There was no significant difference in the Ramsay sedation score between the two groups (*P* > 0.05), as shown in Table 5.

**Table 4:** Comparison of NRS scores between the two groups after extubation.

**Indicator**	**Visceral pain NRS**			**Incision pain NRS**		
**Group**	**Group O (*n* = 30)**	**Group S (*n* = 30)**	***P-*value**	**Group O (*n* = 30)**	**Group S (*n* = 30)**	***P-*value**
1 min	0.9 ± 1.2^a^	2.2 ± 1.2	0.000	1.0 ± 1.2	1.1 ± 0.7	0.893
5 min	1.1 ± 1.2^a^	2.6 ± 1.2	0.000	1.2 ± 1.2	1.2 ± 0.8	0.780
10 min	1.0 ± 1.0^a^	2.9 ± 1.2	0.000	1.2 ± 0.9	1.4 ± 0.9	0.305
30 min	1.1 ± 1.1^a^	3.0 ± 1.4	0.000	1.3 ± 1.0	1.7 ± 0.9	0.140
1 h	1.3 ± 0.9^a^	2.9 ± 1.0	0.000	1.4 ± 0.9	1.8 ± 0.9	0.075
6 h	1.6 ± 0.8^a^	3.3 ± 1.1	0.000	1.7 ± 0.8	2.1 ± 0.8	0.119
24 h	1.8 ± 1.0^a^	3.3 ± 1.2	0.000	1.9 ± 0.9^a^	2.4 ± 0.8	0.038
48 h	1.8 ± 1.0^a^	3.3 ± 1.2	0.000	1.9 ± 0.9^a^	2.4 ± 0.8	0.025

*Compared with Group S*,

a *P < 0.05*.

**Table 5:** Comparison of Ramsay sedation scores between the two groups after extubation.

**Indicator**	**Ramsay sedation scores**		
**Group**	**Group O (*n* = 30)**	**Group S (*n* = 30)**	***P-*value**
1 min	2.7 ± 0.7	2.9 ± 0.9	0.460
5 min	2.5 ± 0.6	2.7 ± 0.8	0.226
10 min	2.1 ± 0.4	2.3 ± 0.6	0.202
30 min	2.0 ± 0.2	2.1 ± 0.3	0.155
60 min	2.0 ± 0.2	2.0 ± 0.0	0.329

## Discussion

The findings of this study were as follows: (1) compared with Group S, the serum IL-6 concentrations of Group O decreased at T3 and T4, and lower than those at T1, while the serum IL-10 concentrations increased; (2) The incidence of PONV and pulmonary inflammation in Group O was lower than that in Group S; (3) The NRS of visceral pain at each time point and incision pain at 6 and 24 h after extubation in Group O was lower than that in Group S.

The NRS score of visceral pain in group O was lower than that in group S at all time points, and the NRS score of incision pain in group O was lower than that in group S at 6 and 24 h after extubation, suggesting that oxycodone has a better analgesic effect than sufentanil, especially for visceral pain, and the analgesic effect lasts longer than sufentanil. After a single intravenous administration of oxycodone, the analgesic duration was 4–5 h. In this experiment, the NRS scores of visceral pain and incision pain in group O were lower than those in group S at 24 and 48 h after extubation, which may be related to oxycodone regulating inflammatory factors *in vivo*. In this study, the incidence of nausea and vomiting in group O is lower than that in group S, suggesting that oxycodone can exert a better analgesic effect, and the incidence of post-operative nausea and vomiting is lower than that of sufentanil. Regarding adverse effects, this study results are consistent with previous research findings. Koh et al. also reported that the rate of post-operative nausea within 1 h after surgery was also significantly lower in the oxycodone group than that in the fentanyl group (15). Wang et al. also reported that the incidences of side effects were comparable between the two groups (16).

There are several research articles (17–20) about the efficacy and safety of using oxycodone for elderly patients' analgesia that have similar findings to our research as well. Compared with common opioids, oxycodone has the advantage of having a better analgesic effect, especially for visceral analgesia, when used for relieving moderate and severe pain, with fewer adverse reactions, stable vital signs and a high level of safety. Elderly patients may often suffer from other chronic pain. In the referenced articles, oxycodone was also used for alleviating chronic pain in the elderly patient, in the form of long-term oral oxycodone capsules (or through a gastrointestinal tube for those who have difficulty swallowing). This makes us wonder whether we can also increase the administration forms of controlled-release oxycodone, or even replace intravenous administration to improve the ease of use of the medication and reduce its cost. There are reports that oxycodone MEAC can act faster when a higher dose is used, and a background infusion of 1 mg^*^h^−1^ can be effective when PCA is injected intravenously after an operation. However, rescue analgesic drugs may still be needed 2 h after the operation (21). The main reason is that it takes a long time to take effect, so it needs to be administered in advance when it is given intravenously. The specific lead time cannot be worked out clearly from this experiment. The exact lead time needs further research.

The incidence of post-operative pulmonary inflammation in group O was lower than that in group S. The concentration of serum IL-6 in Group O was lower than that in Group S and the concentration of serum IL-10 was higher than that in Group S at T3 and 4, and in Group O, the concentration of serum IL-6 at T3 and 4 was lower than that at T1, and the concentration of serum IL-10 was higher than that at T1, suggesting that oxycodone can inhibit the release of serum IL-6, promote the release of serum IL-10, regulate the level of inflammatory factors and reduce post-operative inflammatory reaction. According to previous research, chronic oxycodone administration will also cause a large number of changes in the expression of inflammation/immunity related genes without a bacterial or viral infection (22). This provides a good research direction for us to study the effect of oxycodone on post-operative inflammation in the elderly. In accordance with previous research, we also found that oxycodone can promote the release of anti-inflammatory factor serum IL-10 and inhibit the release of pro-inflammatory factor serum IL-6 after laparoscopic radical gastrectomy in the elderly. This should be for the same reason as the inhibition of TNF-α measured after taking oxycodone in advance (23). The specific mechanism needs to be confirmed by further research.

## Conclusion

In conclusion, the incidence of nausea and vomiting in group O is lower than that in group S, suggesting that oxycodone can exert a better analgesic effect, and the incidence of post-operative nausea and vomiting is lower than that of sufentanil.

### The Limitations of This Experiment

1. Before the experiment, the pain areas of the patients who participated in the experiment were not screened once.
2. A possible influence of the anesthetic drugs on the effects of sufentanil and oxycodone during an operation has not been ruled out, because we still know little about the influencing factors of oxycodone pharmacokinetics (22). Thus, we cannot rule out the possibility of mutual influence.
3. Perioperative nursing and the environment after returning to the ward, which could not be controlled in this experiment, may also have an influence on a patient's pain and inflammation.

## Data Availability Statement

The original contributions presented in the study are included in the article/supplementary material, further inquiries can be directed to the corresponding author/s.

## Ethics Statement

The studies involving human participants were reviewed and approved by Shaoxing People's Hospital. The patients/participants provided their written informed consent to participate in this study.

## Author Contributions

W-lL and Q-lS: conception, design of the research, and writing of the manuscript. Z-mJ: acquisition of data. W-dC: analysis and interpretation of the data. W-lL, Q-lS, and Z-hC: statistical analysis. Z-hC: obtaining financing. Z-hC and X-hZ: critical revision of the manuscript for intellectual content. All authors contributed to the article and approved the submitted version.

## Conflict of Interest

The authors declare that the research was conducted in the absence of any commercial or financial relationships that could be construed as a potential conflict of interest.

## Publisher's Note

All claims expressed in this article are solely those of the authors and do not necessarily represent those of their affiliated organizations, or those of the publisher, the editors and the reviewers. Any product that may be evaluated in this article, or claim that may be made by its manufacturer, is not guaranteed or endorsed by the publisher.

## Abbreviations

ASA-PS: American Society of Anesthesiologists Physical Status
NRS: numerical rating scale
PONV: post-operative nausea and vomiting.
